# Expression Profile of microRNAs Regulating Proliferation and Differentiation in Mouse Adult Cardiac Stem Cells

**DOI:** 10.1371/journal.pone.0063041

**Published:** 2013-05-17

**Authors:** Luis Brás-Rosário, Alex Matsuda, Ana Isabel Pinheiro, Rui Gardner, Telma Lopes, Andreia Amaral, Margarida Gama-Carvalho

**Affiliations:** 1 Instituto Gulbenkian de Ciência, Oeiras, Portugal; 2 Centro de Cardiologia da Universidade de Lisboa, Faculdade de Medicina de Lisboa, Lisboa, Portugal; 3 Center for Biodiversity, Functional and Integrative Genomics, Faculty of Sciences, University of Lisbon, Lisbon, Portugal; Brigham and Women’s Hospital, United States of America

## Abstract

The identification of cardiac cells with stem cell properties changed the paradigm of the heart as a post mitotic organ. These cells proliferate and differentiate into cardiomyocytes, endothelial and vascular smooth muscle cells, providing for cardiac cell homeostasis and regeneration. microRNAs are master switches controlling proliferation and differentiation, in particular regulating stem cell biology and cardiac development. Modulation of microRNAs -regulated gene expression networks holds the potential to control cell fate and proliferation, with predictable biotechnologic and therapeutic applications. To obtain insights into the regulatory networks active in cardiac stem cells, we characterized the expression profile of 95 microRNAs with reported functions in stem cell and tissue differentiation in mouse cardiac stem cells, and compared it to that of mouse embryonic heart and mesenchymal stem cells. The most highly expressed microRNAs identified in cardiac stem cells are known to target key genes involved in the control of cell proliferation and adhesion, vascular function and cardiomyocyte differentiation. We report a subset of differentially expressed microRNAs that are proposed to act as regulators of differentiation and proliferation of adult cardiac stem cells, providing novel insights into active gene expression networks regulating their biological properties.

## Introduction

The observation of cardiomyocyte division first questioned the paradigm that the mammalian heart is a post-mitotic organ. The isolation of adult heart cells expressing markers of stemness (c-kit, Sca-1 or MDR1) displaying the fundamental properties of stem cells: self-renewal, clonogenicity, and multipotency [1] further challenged this paradigm.

It is estimated that approximately 3×10^6^ cells are generated in the human heart every day, arising from the multiplication of cardiac stem cells (CSCs) [2]. In terms of phenotype, CSCs are undifferentiated, keep quiescent until induced to proliferate and may differentiate into one of the three cardiac cell lineages: cardiomyocytes, endothelial cells and vascular smooth muscle cells.

The origin of the CSC population remains unclear. They are either believed to be the progeny of mesenchymal cells from the bone marrow, which homed to the heart through systemic circulation, or to correspond to cellular remnants of the embryonic heart [3]. During embryogenesis, a tightly orchestrated gene expression program involving cardiac specific genes and transcription factors that are activated in succession is initiated, coordinating heart development, along with the differentiation of the main cardiac cell lineages [4]. Interestingly, genes that control cardiac formation during development are active in CSCs. Furthermore, during the differentiation process from CSCs to cardiomyocytes, CSCs seem to replicate the embryonic program [5]–[7]. However, unlike embryological cells developing into cardiomyocytes, for which once the process begins it inexorably leads to the final phenotype, the adult CSC manages to become stuck in an intermediate stage; both the mechanisms that stop and restart CSCs are unknown.

In the last decade microRNAs (miRs) have been found to play important roles in the regulation of multiple biologic functions, including the control of stem cell and tissue differentiation [5], [8]–[11], response to stress and in particular, heart development and disease [12]–[16]. There are approximately a thousand miRs in the human genome, each one targeting multiple RNAs and exerting an influence on their turnover and translation to different degrees, depending on the specific characteristics of the miR-mRNA interaction [17]. As a consequence of these multiple interactions, miRs create complex gene regulatory networks that can serve multiple purposes, from lending robustness to cellular responses, to acting as developmental switches or broad enforcers of tissue and cellular identity [18]–[20]. Therefore, the miR expression profile of a given cell type emerges as a *bona fide* marker of the active regulatory networks that define the cell’s biological characteristics.

The identification of the CSC miR expression profile and of their role in CSC biology has never been systematically addressed. We report the first partial miR expression profile of CSCs isolated from adult mouse hearts, focusing on a subset of miRs known to be involved in the regulation of stem cell and tissue differentiation processes. Comparative analysis with embryonic heart cells from day E9 and immature c-kit positive bone marrow progenitor cells (BMCs) has allowed us to identify a differential expression profile that correlates strongly with the biological properties of this adult stem cell population, providing novel insights into cell identity and phenotype.

## Methods

### CSC Isolation

#### a) Cardiac cell suspension

Balb/c mice were sacrificed by euthanasia with CO2 asphyxiation and the hearts were removed and washed by injecting 1 ml of Hank’s Balanced Salt Solution (HBSS) without Ca2+ e Mg+ (Sigma-Aldrich). The hearts were sliced into small clumps (<1 mm3) in a microtube with 500 ul of F12- Kaighn’s Media (F12-K) (GIBCO #21127) supplemented with 10% Fetal Bovine Serum (FBS - GIBCO), 3% Penicillin/Streptomycin (GIBCO #15140-122) e 1% Transferrin Sodium Selenite (ITSS) (Sigma-Aldrich # 1884) (F12-K+) and incubated at 37°C for 20 minutes with agitation (140 rpm). The tissue suspension was filtered through a 40 µm nylon mesh at least three times in order to exclude the larger cardiomyocytes from the heart tissue. The cell suspension was centrifuged at 12000 rpm for 10 minutes at 4°C and then the pellet was resuspended in 1 ml of FACS buffer (HBSS and 5% FBS).

#### b) Fluorescent activated cell sorting

Cardiac cells were then labeled with monoclonal antibody, anti-Sca1 (δ Mouse LY-6A/E, clone E13–161.7 Rat IgG2a – BD Pharmingen™#553355) conjugated with Fluorescein isothiocyanate (FITC). Staining was done at 4°C in the dark for 30 minutes and washed with FACS buffer. Cells resulting from this procedure were sorted by FACS in a FACS Aria high-speed cell sorter (Becton Dickinson, San José, CA, USA) with a 70 µm nozzle at.5 MPa (70 psi). A 488 nm (15 mW output) coherent Sapphire solid state laser was used to excite FITC. Fluorescein emission was detected with a 530/30 band-pass filter. Sca-1-FITC positive cells were collected directly into an RNA later solution (RNAlater® solution #AM7020-Ambion) and stored at −20°C. Both sample and collection tubes were kept at 4°C throughout sorting.

### Bone Marrow Stem Cell Isolation

#### a) Bone marrow suspension

Isolation of bone marrow lineage negative cells was performed as follows: Balb/c mice were sacrificed by euthanasia with CO2 asphyxiation. The femur and tibia were removed and the bone marrow was collected by flushing with FACS buffer (HBSS and 5% FBS). The cell suspension was filtered through a 40 µm nylon mesh and centrifuged at 12000 rpm for 3 minutes at 4°C. Cells were washed once with FACS buffer and the cell pellet was resuspended with 1 ml FACS buffer.

#### b) Fluorescent activated cell sorting

For Fluorescent Activated Cell Sorting, samples were treated with FcBlock (δ Mouse CD16/32, clone 2.4G2 Rat IgG2b - IGC) for 15 minutes, at 4°C and washed once, stained with an antibody cocktail : B220-Bio (δ Mouse CD45R, clone RA3 6B2 Rat IgG2b - IGC), Mac1-Bio (δ Mouse CD11b, clone M1/70 Rat IgG2b – BD Pharmingen ™#553309), TER-Bio(δ Mouse TER 119/Erythroid cells, clone TER 119 Rat IgG2b – BD Pharmingen ™#553672), GR1-Bio APC (δ Mouse Ly-6G/Ly-6C, clone RB6-8C5 Rat IgG2b – BD Pharmingen™#553125), CD5-PE (δ Mouse CD5, clone 53-7.3 Rat IgG2a – BD Pharmingen™#553023), c-kit-APC APC (δ Mouse CD117, clone 2B8 Rat IgG2b – BD Pharmingen™#553355) for 20 minutes, at 4°C, in the dark and washed twice with FACS buffer. The last staining was done with SAV-PE (δ Mouse Streptavidin-Phyerythrin, clone Streptavidin – BD Pharmingen™#554061) for 20 minutes, at 4°C, in the dark and the cells were washed twice with FACS buffer. Cells resulting from this procedure were sorted by FACS in a BD FACS Aria high-speed cell sorter as above. PE was excited with the 488 nm line, and a 633 nm (18 mW output) JDS Uniphase HeNe air-cooled laser was used for APC excitation. PE and APC emissions were detected using a 585/42 nm and a 660/30 nm band-pass filters, respectively. Cells expressing c-kit (APC positive) and negative for lineage markers (PE negative) were sorted and collected directly into an RNA later solution (RNAlater® solution #AM7020-Ambion) and stored at −20°C. Both sample and collection tubes were kept at 4°C throughout sorting.

### Isolation of Embryonic Heart Cells

For the isolation mouse embryonic hearts cells Balb/c mice were used for embryo collection. Females were sacrificed by cervical dislocation nine days after mating to harvest embryos at the desired stage – E9. Embryos were dissected in PBS buffer and hearts were collected, including the first and second heart field, transferred to RNA later solution (RNAlater® #AM7020-Ambion) and stored at −80°C. RNA extraction and quality control was performed as described.

### RNA Isolation

Total RNA isolation from samples (including miR fraction) was performed with the mirVana miR Isolation Kit (Ambion - #AM1561), following the manufacturer’s instructions. Samples were characterized in the Lab-on-a-chip 2100 Bioanalyzer (Agilent) using the pico6000 RNA chip for quantification and quality control of RNA preservation.

### miR Profiling

The Stem Cell miR qPCR Array with QuantiMir TM (System Biosciences #RA 620A-1) was used to quantify miRs present in the different cell populations by quantitative real-time PCR according to the manufacturer’s instructions on a CFX Real Time System c1000 Thermal Cycler (BioRad) real-time qPCR instrument.

TaqMan miR Assays (Applied Biosystems) were used for quantification of specific miRs and the control housekeeping RNA sno202 using 1 ng of total RNA input as indicated by the manufacturer.

### mRNA Expression Analysis

Removal of genomic DNA from RNA samples was carried out using DNase I (Fermantas). Reverse transcription was done using the RevertAid™ H Minus First Strand cDNA Synthesis Kit (Fermentas) with 1 ug of RNA and oligo dT priming according to the manufacturer’s instructions.

The PCR amplification of the cDNA products was performed with the DreamTaq™ DNA Polymerase (Fermentas). The following primers were used for RT-PCR analysis: GAPD - Forward primer: TGCACCACCAACTGCTTAGC; Reverse primer: GGCATGGACTGTGGTCATGAG; c-Myc - Forward primer: TGAGCCCCTAGTGCTGCAT; Reverse primer: AGCCCGACTCCGACCTCTT; Isl1 - Forward primer: TATCCAGGGGATGACAGGAA; Reverse primer: TTGACCAGTTGCTGAAAAGC.

### Data Analysis

Analysis of qPCR array data was performed as follows: raw Ct values were normalized by the quantile method using HTqPCR package (Dvinge and Bertone, 2009) implemented in Bioconductor (Gentleman et al. 2004). The quantile method produces a uniform empirical distribution of Ct values across samples, allowing for direct comparison of Ct values, and is especially useful when housekeeping genes show significant variation across samples, as was the case in this study. miRs which were outside the 90% CI were considered unreliable and miRs which had a Ct>38 for more than 3 libraries were considered undetermined and were not considered for further analysis. miRs with relatively constant Ct levels are less likely to be differentially expressed, so including them in the downstream analysis would cause some loss of power when adjusting the p-values for multiple testing (the miR-by-miR hypothesis testing). Variation across samples was assessed using the interquantile range values (IQR) for each miR. All miRs with an IQR below 1.5 were filtered out.

Clustering analysis was performed by estimating the euclidean distance between samples using gplots package of R (http://cran.r-project.org/web/packages/gplots/index.html).

Analysis of differential expression was performed using the lmfit function of the Limma package of Bioconductor (Smyth, 2004). The Limma package applies a linear model to the expression data for each gene and tests for significant differences using a moderated t-test based on an empirical Bayes method to moderate the standard errors of the estimated log-fold changes. This results in more stable inference and improved power, especially for experiments with small numbers of replicates. p values were adjusted with the Benjamini-Hochberg algorithm to control for false discovery rate. microRNAs with an adjusted p value<0.01% were considered to be differentially expressed.

### Ethics Statement

All animal work was performed according to Portuguese laws and European Union Directives. Mice were exclusively used for collection of sample tissues after being sacrificed according to standard procedures.

## Results

### The miR Expression Profile of Adult Mouse CSCs Reveals Commitment to Cardiac Lineages

The characterization of the miR expression profile of a specific cell type is expected to provide relevant insights into the regulatory networks that define its biological properties. However, profiling transcript levels from rare cell populations with low total RNA levels can represent a technical challenge. CSC isolation has been reported by several laboratories worldwide, using protocols that include tissue digestion and Fluorescence Activated Cell Sorting (FACS) with stem cell membrane markers as c-kit, Sca-1 or MDR1 [2], [21], [22]. However, previous studies on CSC gene expression have focused either on embryonic cells [23] or on fetal cells after proliferation in culture [24].

To obtain high quality total RNA samples in order to characterize the adult CSC miR expression profile, we developed a method to isolated cells from mice hearts by soft mechanical destruction of cardiac tissue, followed by Sca-1 antigen labeling and FACS sorting (Figure S1). Cells isolated through this approach could be grown in culture and induced to proliferate or differentiate into spontaneously beating cardiomyocytes, as expected from *bona fide* cardiac progenitors (data not shown). As control, we isolated bone marrow c-kit+ lineage- BMCs from the same mice. Quantifiable amounts of high quality total RNA could be obtained from sorted 10^5^ BMCs, while total RNA from 4×10^5^ CSCs remained below detection limits (50 pg/µl). However, using RT-qPCR we could consistently detect the expression of the housekeeping sRNA sno202 (not shown).

The abundance of total RNA is known to vary widely across tissues and, in particular, between proliferating and quiescent cells [25]. The low levels of total RNA in CSCs impose significant restrictions to large scale profiling studies without the use of bias-prone pre-amplification steps. However, highly sensitive, qPCR based approaches may be applied under such conditions. We therefore generated a focused profile of the expression of 95 miRs involved in stem cell maintenance and differentiation processes in mouse CSCs using a commercial RT-qPCR array (see Table S1) from three independent samples. Data from these samples showed a good correlation after normalization, with one third of the miRs analyzed being below detection limit. The most abundant miRs detected - Table 1 – are all known to have strong functional correlations to cardiovascular development and disease. In line with the known potential of CSC to give rise to distinct cardiac lineages, the most abundantly expressed miRs found in these cells are considered specific of cardiomyocyte, endothelial and vascular smooth muscle cells, revealing possible regulatory mechanisms that underlie the CSC differentiation ability.

**Table 1:** Top 10% expressed miRs in adult CSCs.

miR	Av expr (Ct)	Reported function	Reference
miR-125b	28.6±0.2	Involved in cardiac stress response.	[48]
miR-126	29.2±1.0	Regulator of endothelial lineage and angiogenesis in cardiovascular development. Involvedin cardiac disease and adult cardiomyocyte hypertrophy.	[49]
miR-133a	29.4±0.6	Regulation of cardiomyocyte differentiation. Promotion of undifferentiated progenitor expansion. Involved in cardiac disease and adult cardiomyocyte hypertrophy.	[49]
miR-23a	29.7±0.3	Response to cardiac injury.	[48]
miR-24	29.9±0.8	Response to cardiac injury.	[48]
miR-23b	30.1±0.4	Response to cardiac injury.	[48]
miR-125a	30.1+0.5	Response to cardiac injury.	[48]
miR-30c	30.1+0.3	Involved in cardiac disease and adult cardiomyocyte hypertrophy.	[49]
miR-132	30.3+0.8	Regulators of endothelial lineage and angiogenesis in cardiovascular development.	[31]

### Comparative Profiling of CSC, Embryonic Heart Cells and Adult BMCs Reveals a Cardiac Stem Cell Specific Signature

In order to further identify key miRs regulating CSC biology, we performed a comparative approach with two populations of multipotent progenitors that may give rise to cardiac cell lineages and eventually represent the precursors of stem cells isolated from the adult heart: immature bone marrow cells (BMCs; c-kit+, hematopoyetic lineage negative) and embryonic heart cells from day E9.

The comparison between these populations is expected to reveal some underlying differences regarding the activation of gene expression programs related to stem cell maintenance, proliferation and cardiac lineage commitment, providing important insights into the origins and biology of adult cardiac stem cells.

**Figure 1:**
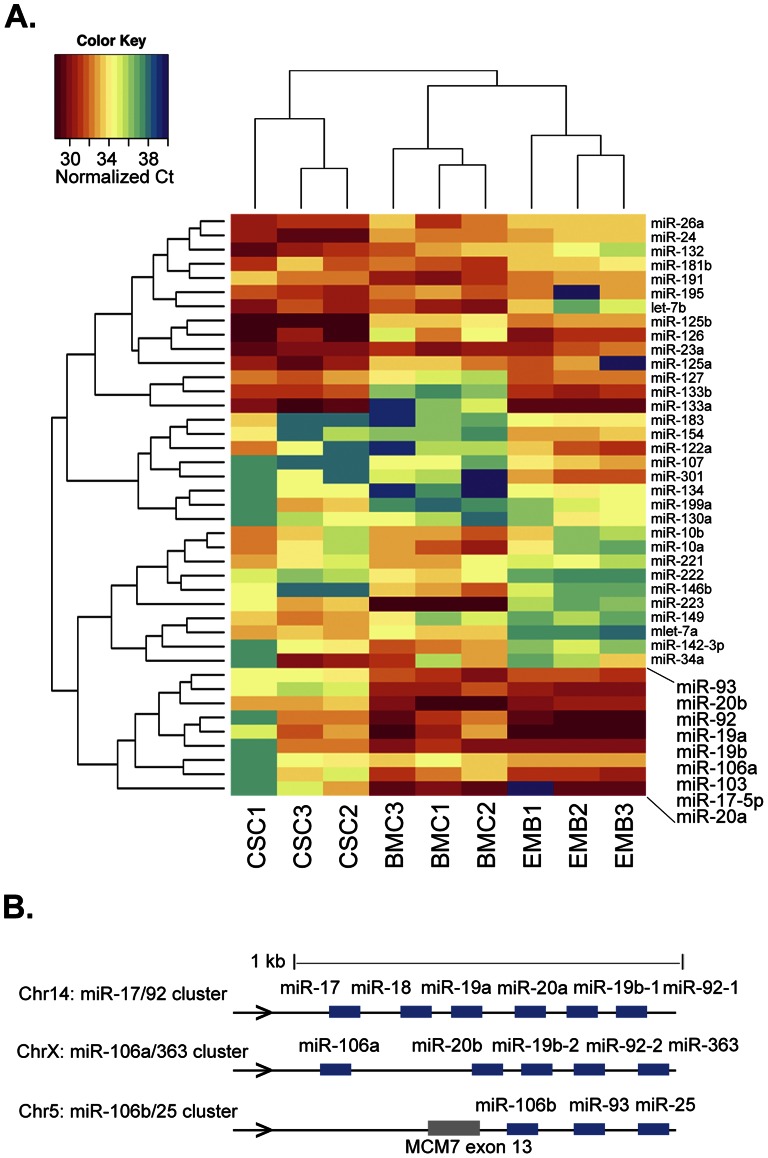
Comparative miR expression in progenitor cell populations with cardiogenic potential. **A.** Hierarchical clustering for samples and miRs of three independent replicates of mouse CSCs (CSC), adult BMCs isolated from mouse bone marrow (BM) and mouse embryonic heart day E9 (EMB). Box highlights the miR branch separating CSCs from BMCs and embryonic heart cells, composed almost exclusively of miR-17/92 family members. Data shown refers to normalized Ct values (color key) of filtered subset of 41 differentially expressed miRs, out of the 95 miRs analyzed (see methods). **B.** The mouse miR-17/92 family loci. Expression levels for all members of this cluster were determined, with the exception of miR-363, which was not present in the PCR array used in this study.

Total RNA samples isolated from three independent replicates of mouse BMCs and E9 embryonic hearts were used for miR profiling with the Stem Cell miR q-PCR array (see Table S1). To obtain an overview of the similarities and differences of their miR expression profile relative to CSCs, we performed an unsupervised clustering analysis of our expression datasets for both sample and miR, after normalizing miR Ct values across samples with the quantile method and filtering out miRs with low variation across all samples (Fig. 1).

The biological replicates of each cell type clustered together, demonstrating the robustness of this dataset. Surprisingly, CSCs segregated independently from both BMCs and embryonic heart samples, which are grouped in a common branch. Analysis of the clustering profile suggested that this was mainly determined by a group of nine miRs that is highly expressed in these two cell populations and absent from CSCs (Fig. 1A, inset). Eight of these miRs belong to the miR-17-92 cluster family, encoded in three related genomic loci (Fig. 1B). The miR-17-92 cluster family is highly expressed in proliferating cells and has been shown to have oncogenic potential [23], [26], [27]. Additionally, they have been shown to be required for normal heart development [28]–[30] and, more interestingly, to control the differentiation of cardiac progenitors [6], [28], [31].

### CSCs Differentially Express Key Regulators of Cardiac and Vascular Development

**Figure 2:**
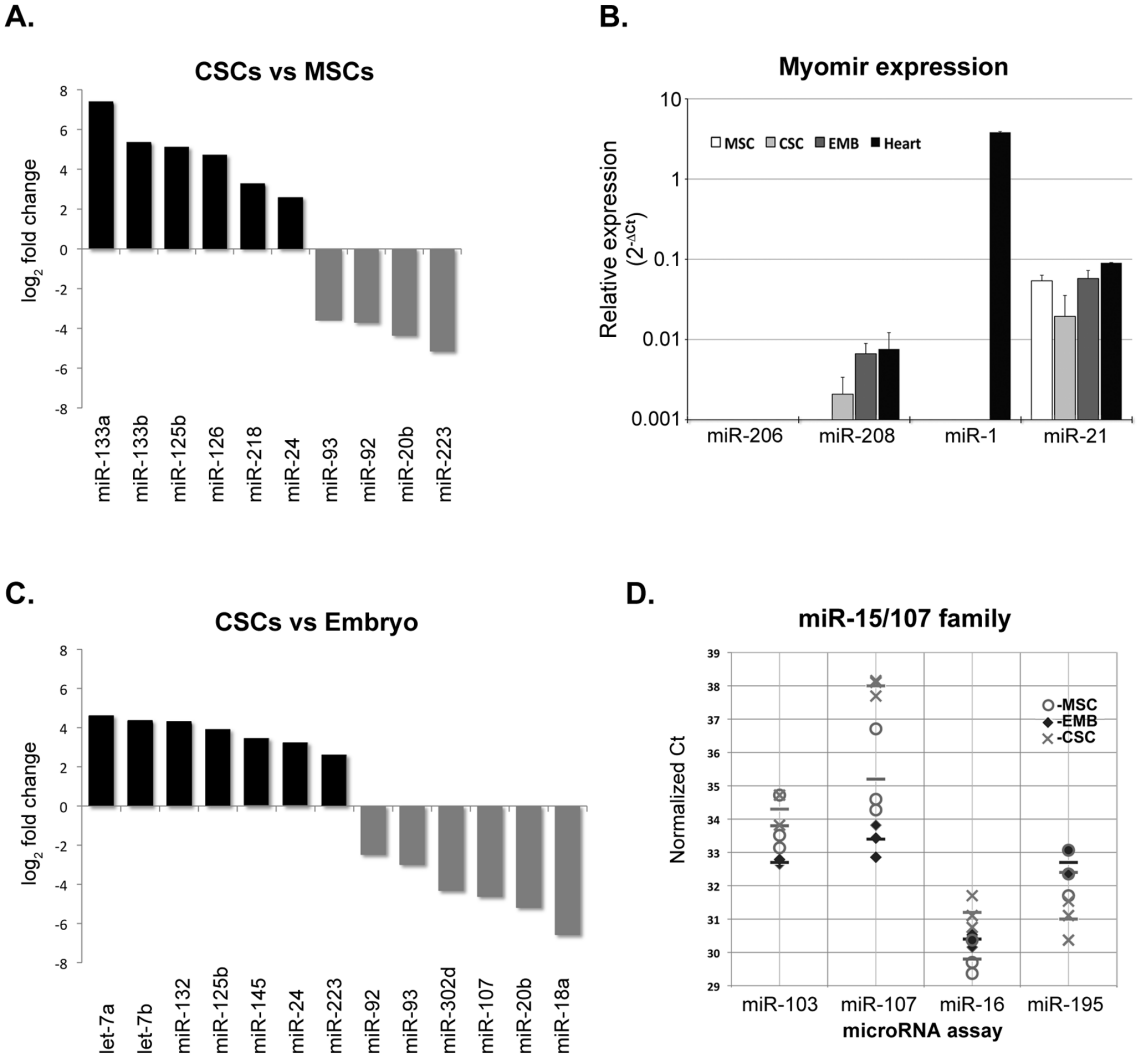
Differential expression analysis of microRNAs. **A.** Differentially expressed miRs in adult cardiac and immature bone marrow stem cell populations (CSCs and BMCs). Average log2 expression ratio of all differentially expressed miRs (p<0.01). **B.** Comparison of expression of muscle specific miRs in embryonic heart cells (EMB), BMCs and CSCs, and mouse heart tissue using Taqman probes (n = 3). Data shows relative expression (2^−ΔCt^) normalized to sno202 RNA expression levels. **C.** Differentially expressed miRs in adult cardiac versus embryonic mouse heart cells. Average log2 expression ratio of all differentially expressed miRs (p<0.01). **D.** miR-15/107 family expression levels in the cell populations characterized in this study. Plot displays normalized Ct values for each independent biological replicate sample of miR-15/107 family members present in the qPCR array. miR-107 is specifically downregulated in CSCs. Horizontal bar marks average Ct.

The clustering analysis presented above (Fig. 1) additionally reveals groups of miRs that distinguish CSCs from both BMCs and embryonic heart cells. In order to understand how CSCs differ from BMCs we performed a differential expression analysis between the two samples and identified ten differentially expressed miRs (adjusted p value<0.01), six of which are up-regulated in CSCs (Fig. 2A). As expected, three members of the miR-17-92 family already identified in the clustering analysis were found to be significantly down-regulated in CSCs, together with miR-223, a well-known regulator of hematopoietic differentiation [32]–[34]. Among the six up-regulated miRs in CSCs we find the four most highly expressed miRs in these cells: miR-125b, miR 126, miR-133a and miR-24. miR-133a emerged as the most differentially expressed between the two cell types, with an 100 fold higher expression level in CSCs, followed by a member of the same miR family, miR-133b. These muscle-specific miRs are key regulators of the cardiomyocyte differentiation process and have been shown to control the expression of cardiomyocyte specific proteins (reviewed by [13], [35], [36]). The other three miRs also have been linked to cardiac functions (see discussion). Additionally, miR-218 also displays a significant enrichment in CSCs when compared to BMCs. miR-218 was recently shown to function in vascular development [37]–[39] and to be required for heart tube formation [28], [39], [40]. This CSC miR expression profile implies that CSCs already display a significant degree of commitment to cardiac lineages. To further explore this aspect and discard the possibility of eventual contamination of our samples with differentiated cardiomyocytes, we independently determined the expression levels of miR-1, a hallmark of cardiomyocyte differentiation, and two additional miRs known to be essential for muscle differentiation processes: miR-208a and miR-206. miR-208a is encoded in an intron of the myosin heavy chain gene Myh6 [41], [42] and is part of a family of so-called ‘myomirs’, named after their important roles in muscle differentiation. miR-206 is another member of the miR-1/133 family that exhibits skeletal muscle specific expression. For this purpose, we used commercially available Taqman based RT-qPCR probes, and compared their expression levels in mouse heart tissue. As a control, we quantified the expression of the abundant and ubiquitously expressed miR-21. This analysis confirmed our previous observation that miR-1 cannot be detected in adult CSCs or in embryonic heart cells at day E9, although it is highly expressed in mouse heart tissue (Figure 2B). In contrast, the expression levels of mir-208a are very similar in both cell types and heart tissue, albeit undetectable in BMCs, confirming the commitment of CSCs to the cardiomyocyte lineage. Interestingly, this is in agreement with the identification of cardiac myosin heavy chain-alfa in a proteomic profiling of CSCs (Brás-Rosário, unpublished data). As expected, miR-206 could not be detected in any of the samples tested. These results confirm that CSCs already display a significant degree of commitment to cardiac lineages, displaying a miR expression profile that is more similar to embryonic cardiac progenitors.

### Overexpression of Anti-proliferative miRs Distinguishes Cardiac Stem Cells from Embryo Heart Cells

In order to better understand the similarities and differences between CSCs and embryonic heart cells, we performed a differential expression analysis between the two datasets and found a total of thirteen miRs with either significant up-regulation (seven miRs) or down-regulation (six miRs) (adjusted p value<0.01) (Figure 2C). Similar to what was observed when comparing cardiac and immature bone marrow SCs, four of the six down-regulated miRs belong to the miR-17∼92 cluster family, corresponding to some of the most highly expressed miRs in embryonic heart cells.

Of the other two down-regulated miRs in CSCs, miR-302d has been described as embryonic stem cell specific in both mouse and humans [43], [44], whereas miR-107 belongs to the miR-15/107 family. In mouse this family includes 11 miRs that share the seed sequence AGCAGCA and are encoded by independent genes [5], [21], [45]. The miR-15/107 family has been shown to co-regulate several mRNA targets that encode key proteins for distinct cellular functions, including metabolic regulation, cell cycle control, control of metastasis and epithelial mesenchymal transition and stem cell plasticity [5]. To understand if the observed down-regulation of miR-107 may reflect a CSC specific fine-tuning of the pathways regulated by this group of miRs, we looked for the presence of other members of the miR-15/107 family in our dataset. Four miRs, representing the most highly expressed members of this family (miR-16, miR 103 and miR-195), were present in the array used in our study. Strikingly, they all show similar expression levels in embryonic heart, BMCs and CSCs, with the exception of miR-107 (Figure 2D). Thus, down regulation of miR-107 seems to be a distinctive feature of CSCs, implying a CSC specific regulatory profile for miR-15/107 target genes. The implications of this are presently unclear, as miR-107 seems to share almost 80% of its targets with both miR-16 and miR-103 and there are contradictory reports regarding its role, for example, in cell cycle control [5], [9]. Interestingly, this family of miRs was recently linked to the control of the post-natal mitotic arrest of cardiomyocytes [12], which seems to involve the up-regulation of several miR-15 family members, while miR-107 becomes down-regulated. These results point to a possible link between the expression of this miR and the maintenance of a quiescent state in CSCs.

In addition to this group of down-regulated miRs, CSCs can be distinguished from embryonic heart cells on the basis of seven up-regulated miRs: let-7a, let-7b, miR-24, miR-125b, miR-132, miR-149 and miR-223 (Fig. 2C).

As mentioned above, miR-24, miR-125b and miR-132 are among the top expressed miRs in CSCs. In contrast, we observed that miR-223 is down regulated in CSCs in comparison to BMCs, in agreement with its established role as a regulator of hematopoyesis. Little is known regarding the function of miR-149, in addition to the fact that it has been identified as being down regulated in response to cardiac injury [18].

The let-7 miRs belong to a conserved family of genes known to be involved in the control of stem cell proliferation and differentiation [21], acting as tumor suppressors. Interestingly, let-7a targets the oncogenic transcriptions factors Ras and Myc and is negatively regulated by the latter. Thus, high levels of let-7a/b expression are in agreement with the non-proliferating phenotype of CSCs, which seem to have several Myc-dependent expression networks silenced. miR-24 is also a known anti-proliferative miR that acts as a negative regulator of Myc expression [26].

### Silencing of myc-dependent Pathways in CSCs

**Figure 3:**
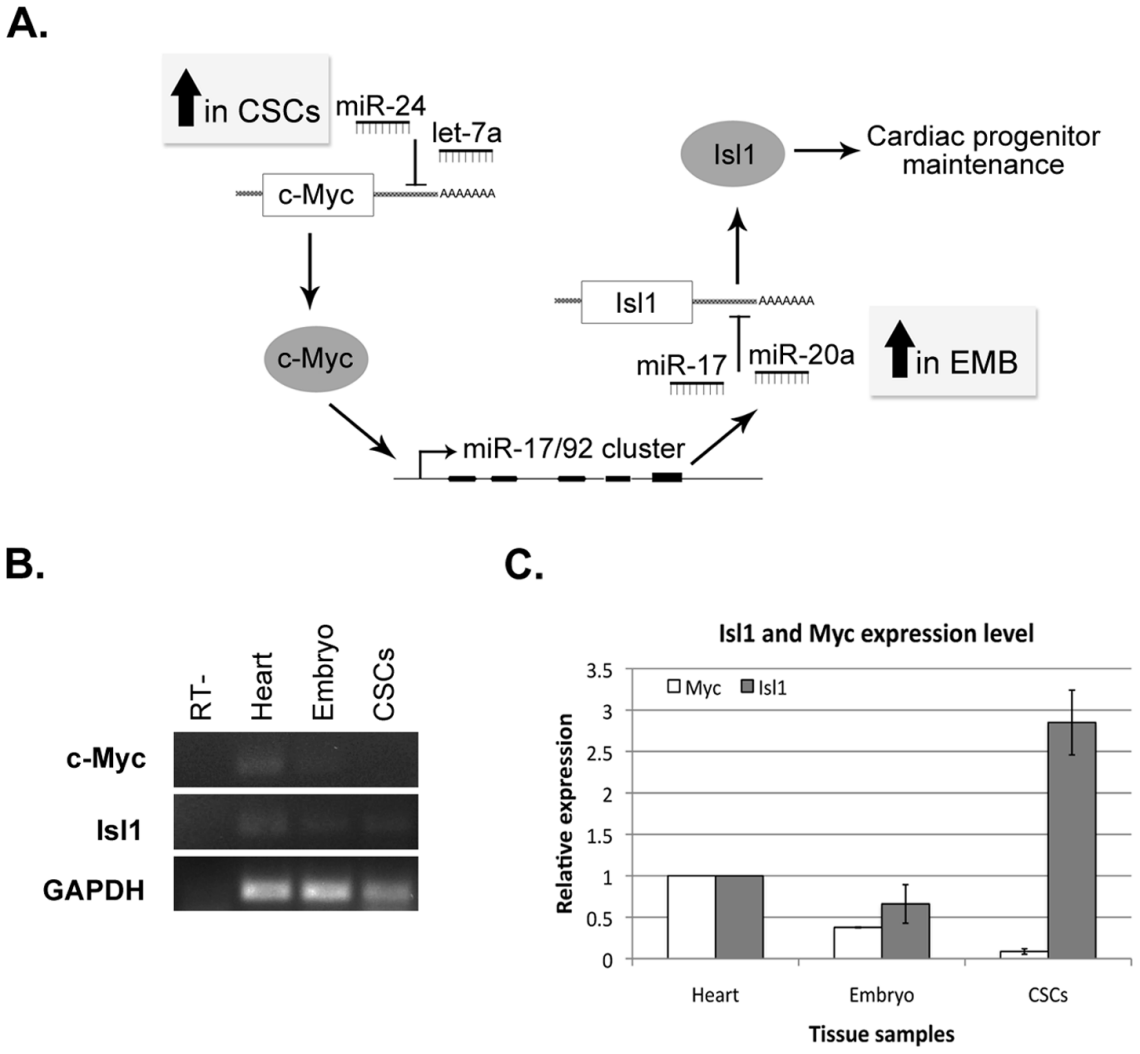
Expression of myc and Isl1 in CSCs. **A.** Proposed regulatory network involving differentially expressed miRs in embryonic heart (EMB) and CSCs. **B.** Expression of c-Myc and Isl1 in embryonic heart and CSCs by semi-quantitative RT-PCR. Results are representative of three independent replicate samples. **C.** Quantification of relative expression levels of c-Myc and Isl1 (n = 3). Expression levels are normalized to GAPDH.

The comparison of miRs expressed in CSCs and embryonic cardiac progenitors suggests that these cell populations differ in the activation of Myc dependent pathways. Thus, CSCs over-express miR-24 and the let-7a/b miRs, which are known to negatively regulate Myc expression, while showing a very strong down-regulation of the miR-17/92 family, which is a known transcriptional target of Myc. Interestingly, this miR family was recently shown to be required for the terminal commitment of embryonic cardiac progenitor cells from the secondary heart field to cardiomyocyte differentiation [28]. This involves the direct targeting of the mRNA encoding the LIM-homeodomain transcription factor Islet1 (Isl1), a marker of pluripotent undifferentiated cardiovascular progenitors, which has been proposed to act as an anti-differentiation factor. Together with our miR profiling data, these observations led us to propose a network of transcriptional interactions that could underlie the CSC phenotype (Fig. 3A). We therefore decided to look at the expression of c-Myc and Isl1 in our Sca1+ CSC populations and compared it to embryonic cardiac progenitors and total heart tissue (Fig. 3B). Interestingly, we find that, as predicted, c-myc expression is silenced in CSCs whereas Isl1 expression can be detected at relatively high levels when compared to embryonic progenitor cells. Our results suggest that Isl1 may indeed represent a common marker of cardiac progenitor cells, and that the activation of the miR-17-92 cluster may act as a general orchestrator of cardiac progenitor differentiation.

## Discussion

In this study we present a focused profiling of the expression of stemness and differentiation related miRs in Sca1 positive adult CSCs. Our results provide new insights into active regulatory networks underlying the control of proliferation and differentiation, two critical aspects of CSC biology.

In spite of the its importance for the understanding of regulatory mechanisms controlling CSC function, the characterization of transcript abundance in this cell population is complicated by three factors: the rarity of these cells (less that 2% of the total heart cells [6]); the difficulty of isolating good quality samples from fibrous heart tissues, and the comparatively low levels of total mRNA present in these cells. Indeed, while we where easily able to quantify and evaluate the quality of total RNA isolated from 10^5^ Sca1+ BMCs isolated from the bonemarrow (Fig. S1), total RNA obtained from four times as much Sca1+ CSCs remained below detection limits (i.e, 50 pg/µl using the Agilent RNA 6000 Pico Kit), in spite of significant improvements in purification procedures. This imposes significant restrictions to the application of large scale profiling studies, while allowing for the use of focused, highly sensitive, qPCR based miR-profiling approaches.

The consistent identification of miRs that are considered specific of the three cell lineages that CSCs can differentiate into - cardiomyocyte, endothelial and vascular smooth muscle cells ([32], see discussion below) - underscores the robustness of our isolation procedure. Of note, endothelial pericytes may also express Sca-1 [35]. However, the degree of enrichment in cardiomyocyte specific miRs that we observed makes it unlikely that a significant amount of these cells may be present in our samples. The fact that we could not detect expression of the cardiomyocyte-specific miR-1 in CSC samples using two independent methods, while we confirmed it to be highly expressed in total heart tissue, further demonstrates the absence of significant contamination from other cardiac cell types. Therefore our profiling study specifically reflects the miR-regulatory networks underlying CSC function and cell fate decision.

The comparison of CSC miR expression profile with that of BMCs and embryonic heart cells, which display similar differentiation potential and have both been hypothesized to correspond to the CSC progenitor population [37], gives unprecedented information on the possible miR regulated pathways involved in establishing the CSC phenotype. Interestingly, silencing of the miR-17/92 cluster emerges as the major difference between CSCs and their two potential progenitor populations. In heart development, this cluster is activated by BMP signaling to downregulate the cardiac progenitor genes Isl1 and Tbx1 and promote cardiac differentiation in a feed forward mechanism [28]. Therefore, the miR-17-92 cluster seems to play an essential role in controlling the timing and differentiation potential of Isl-1 positive cardiac progenitor cells, which represent a common multipotent progenitor lineage present both in the embryonic and adult heart, with the ability to generate both cardiomyocyte, endothelial, and VSMC descendents [4], [46], [47].

The miR-17-92 cluster is known to be regulated by c-myc leading us to hypothesize that the expression of this transcription factor is down-regulated in CSCs. In agreement with this model, we were unable to detect c-myc transcrips in CSCs, in contrast with what was observed in samples from adult and embryonic heart. In parallel with the down-regulation of this gene, and in contrast with previous studies [6], [7], we detected a significant expression of the Isl1 transcription factor. This discrepancy may be explained by a higher quality of the CSCs RNA samples used in the present study, and suggests that Isl1 may indeed represent a common marker of all cardiac progenitors, ending a long-term unexplained discrepancy regarding Sca1+ cells [8], [10], [11].

In addition to the down-regulation of the miR-17-92 cluster, the differential expression analysis of CSCs in comparison with BMCs and embryonic cardiac cells identified the over-expression of several miRs involved in the control of cellular differentiation processes. The expression of miR-133a, miR-133b and miR-208 is considered a specific mark of cardiomyocyte lineage differentiation [13]–[16]. In this study, we have found that these miRs are highly expressed in CSCs, while expression of miR-1, the other cardiomyocyte specific miR, could not be detected. The muscle specific miR-1 and miR-133a are encoded in the same bicistronic transcriptional unit, under the control of the cardiogenic transcription factors MEF2 and SRF [19], [20]. Both miRs have been shown to be involved in the control of the expression of cardiac specific proteins [2], [22]. Additionally, miR-1 and miR-133a are key regulators of cardiomyocyte proliferation and differentiation, assuming antagonistic roles in these processes. Indeed, while miR-1 promotes differentiation of ES cells towards a cardiac fate, miR-133 inhibits differentiation into cardiac muscle [23], [27]. The differential expression of miR-133 in CSCs is therefore clearly indicative of a commitment to the cardiomyocyte lineage, compatible with the maintenance of an undifferentiated, non-proliferative state. The parallel identification of miR-208 expression, an intronic miR encoded in the cardiac alfa-myosin heavy chain (αMHC) gene, which promotes the transition from the embryonic to adult isoforms of cardiac contractile proteins, further stresses the CSC commitment to the adult cardiomyocyte lineage. Balancing the CSC commitment to the cardiomyocyte lineage is the identification of a significant overexpression of miR-126, considered the only miR characteristic of endothelial cells [29], [30]. Some of the other miRs identified as highly expressed in CSCs have also been specifically implicated in endothelial cell development and regulation. In particular, miR-132 is an inducer of endothelial cell differentiation and proliferation, pointing to the commitment of CSCs to this lineage [28], [31]. In addition to the above mentioned role as a regulator of endothelial differentiation, miR-132 was recently shown to regulate the expression of the β2 subunit of the cardiac L-type calcium channel protein [33], [34] and to function as a paracrine activator of heart healing [13], [36]. miR-23b is involved in the mechano-transduction of proliferative signals in endothelial cells [38], [39], whereas miR-218 was recently shown to be a critical regulator of vasculogenesis and heart tube formation during development [39], [40]. One should also remark that both miR-126 and miR-218 interact with the VEGF pathway, raising the hypothesis that VEGF is involved in CSCs differentiation into endothelial cells. Finally, CSCs are also known to differentiate into a third lineage, vascular smooth muscle cells (VSMCs). VSBMCs display an unusual ability to switch between a proliferative and differentiated (contractile) phenotype ant it was recently shown that miR-24 is involved in the regulation of this process, promoting VSMC dedifferentiation [41], [42]. Additionally, miR-24 was shown to suppress cardiomyocyte apoptosis [43], [44].

These results suggest that CSCs display a significant degree of commitment to differentiation into the three main cardiac lineages - cardiomyocyte, endothelial and vascular smooth muscle cells - and provide evidence for the presence of miR-dependent regulatory networks defining an overall cardiac-committed yet undifferentiated phenotype.

An adult stem cell population needs to maintain its numbers, control the balance between cell division and the number of cells that undergo differentiation into diverse cell lineages. Comparative profiling of CSCs and embryonic heart cells identified the let-7 miR family members, let-7a, let-7b, miR-125a and miR-125b, as well as miR-223 as being upregulated in CSCs. Additionally, another member of the let-7 family, miR-125a, was found to be highly expressed in CSCs. These miRs have reported roles as regulators of stem cell proliferation, apoptosis and differentiation, and are likely to contribute to the maintenance of a non-proliferative and undifferentiated state, albeit with limited potential [5], [21], [45].

All together, the miR expression profile of CSC conveys a picture of tight control of proliferation and cell fate orientation with three cardiac lineages – cardiomyocyte, endothelial cell and vascular smooth muscle – in accordance with previous known biological activities of CSCs, reinforcing the possible role of miRs in their control. A major question that remains to be answered is whether the Sca-1 positive cells characterized in this study represent a homogenous population where these regulatory networks are all active, or whether specific subtypes within this population differentially over-express markers of each lineage and therefore display a more defined commitment state than what may be inferred from the analysis of the whole population. Future studies will help address this question and explore the role of specific miRs identified in this work in the control of CSC function.

## Supporting Information

Figure S1 **Fluorescence activated cell sorting of Sca-1 expressing cells isolated from adult mouse heart.** An unstained sample (left plot, A) was used as negative control to define the sorting gate. Sca-1 positive cells (right plot, A) were identified based on FITC-positive signal in the 530/30 nm channel, and distinguished from negative cells with high autofluorescence using an autofluorescence channel with 585±20 nm range.
(TIF)

Table S1 Raw and normalized qPCR data for miR expression profiles in mouse adult cardiac stem cells (CSC), bonemarrow lin negative, c-kit positive immature bone marrow stem cells (BM) and mouse embryonic heart (EMB). Excel file, two worksheets.
(XLS)
