# Supplementary figures and images for: Expression Profile of microRNAs Regulating Proliferation and Differentiation in Mouse Adult Cardiac Stem Cells
Source: PLoS One. 2013 May 17;8(5):e63041. doi: 10.1371/journal.pone.0063041 (PMC3656880; doi:10.1371/journal.pone.0063041)

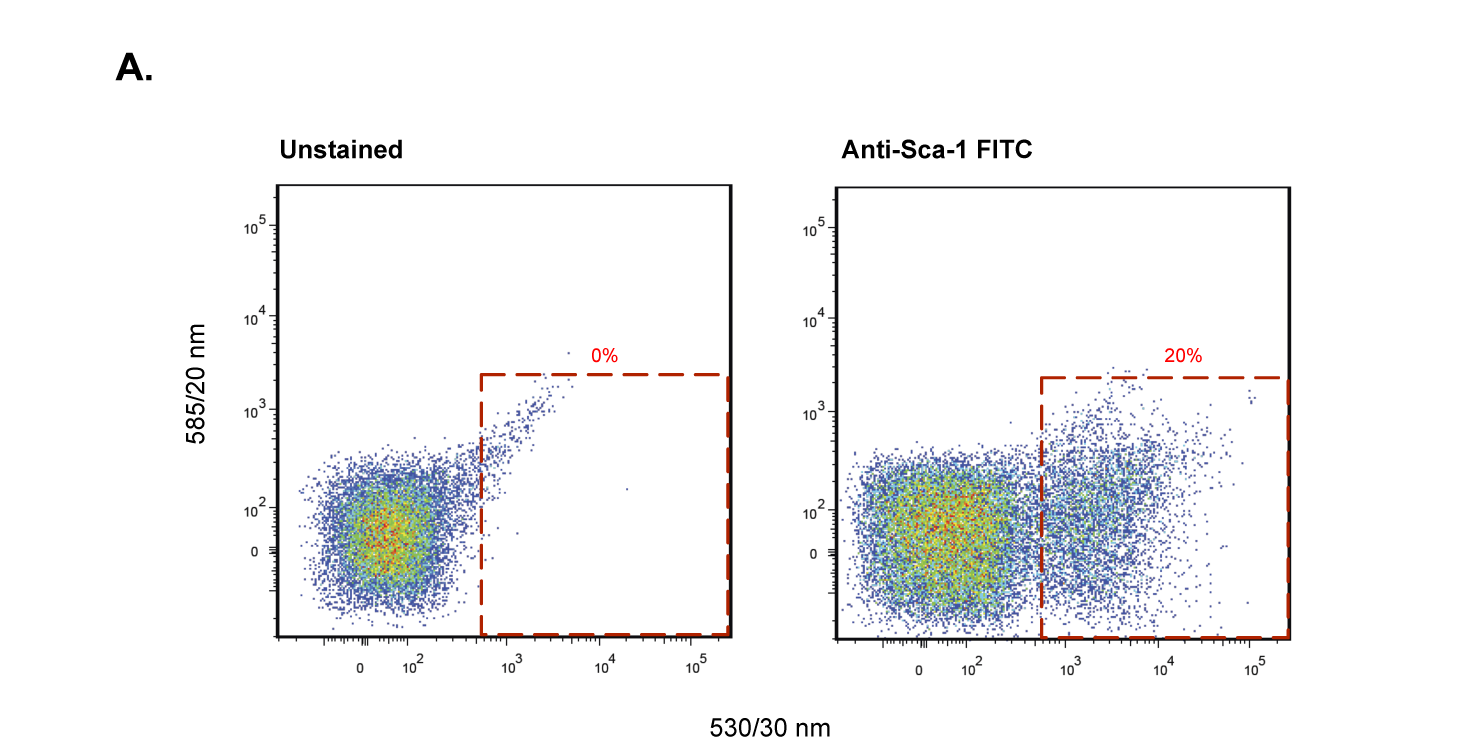

Supplement: Figure S1 — Fluorescence activated cell sorting of Sca-1 expressing cells isolated from adult mouse heart. An unstained sample (left plot, A) was used as negative control to define the sorting gate. Sca-1 positive cells (right plot, A) were identified based on FITC-positive signal in the 530/30 nm channel, and distinguished from negative cells with high autofluorescence using an autofluorescence channel with 585±20 nm range. (TIF) [file pone.0063041.s001.tif]
